# Beyond the bedside: A scoping review of the experiences of non‐practising health care professionals in Health Professions Education

**DOI:** 10.1111/medu.15529

**Published:** 2024-09-12

**Authors:** Helen R. Church, Megan E. L. Brown, Lynelle Govender, Deborah Clark

**Affiliations:** ^1^ Faculty of Medicine and Health Sciences University of Nottingham Nottingham UK; ^2^ School of Medicine Newcastle University Newcastle UK; ^3^ Division of Anatomical Pathology, Faculty of Health Sciences University of Cape Town Cape Town South Africa; ^4^ Division of Clinical Medicine, School of Medicine and Population Health, Faculty of Health University of Sheffield Sheffield UK

## Abstract

**Introduction:**

The shortage of educators within Health Professions Education (HPE) threatens the optimal training of the future health care workforce. Furthermore, without recruitment of diverse and skilled faculty, targets to expand the workforce will not be possible. Non‐practising health care professionals offer extensive knowledge and qualifications within health care, without the competing clinical commitments of their clinical academic colleagues, and therefore are ideally positioned to support education and training initiatives. However, the limited available evidence suggests that these individuals face significant challenges transitioning from clinical to academic roles. The purpose of this scoping review is to address the research question ‘What is known about the career experiences of non‐practicing healthcare professionals (defined as individuals with clinical backgrounds who no longer engage in direct patient care) across various professions and internationally, within the field of health professions education?’. To do so, we aim to map the global experiences of non‐practising health care professionals from different specialties and disciplines transitioning to HPE, with a view to both current support strategies that aim to recruit and retain these individuals and fuel future research in this area.

**Methods:**

Following Arksey and O'Malley's scoping review guidelines, a research question was formulated focussing on exploring the career experiences of non‐practising health care professionals now working in HPE. Searching seven literature databases and grey literature identified 51 articles for analysis. Both quantitative and qualitative methods were utilised to chart and thematically analyse data to identify key themes.

**Results:**

There has been a rise in publications on this topic, with most studies originating from the United States and focusing on nursing. Transition to academia is marked by significant challenges, including identity shifts, renumeration and professional progression tensions, licencing issues and financial concerns. Support systems are crucial to navigating new roles alongside personal/professional development but often lacking.

**Discussion:**

This scoping review highlights challenges and opportunities for non‐practising health care professionals in HPE. Additional support for making the transition to education, including structured onboarding processes and long‐term mentoring relationships, would be beneficial. Recognising the liminal space these professionals occupy might also facilitate more effective integration into academic roles, contributing to a more dynamic and inclusive HPE environment. Future research should explore these experiences from broader professional and geographical perspectives and employ an intersectional approach to fully understand and support this growing demographic in our field.

## Abstract 2

.@helenrchurch et al. summarise the current evidence pertaining to clinician educators becoming non‐practicing, detailing how we can support them during this career move.

## 1 INTRODUCTION

In response to a critical global health care workforce shortage, the World Health Organisation's^1^ Workforce 2030 strategy sets ambitious targets aimed at improving the density and distribution of health professions graduates. Achieving these goals requires a robust Health Professions Education (HPE) system supported by diverse, professionally trained faculty, including basic scientists, educationalists and health care professionals in educator roles.^2^ However, although the demand for expertise in HPE is escalating^3^ (due to, e.g. increasing student enrolments^4^), the career path for health care professionals interested in becoming full‐time educational faculty remains a path less travelled and even less discussed in the literature.

Health care professionals interested in leaving their clinical roles for academic positions within HPE (a group that has, elsewhere, been termed ‘non‐practicing’ health care professionals^5^) represent a population with great potential in helping to address workforce shortages within HPE. These individuals can play vital roles in education, including delivering teaching and undertaking operational tasks (admissions, assessment, etc.), curricula development, research and leadership. As greater numbers of health care professionals are choosing to leave their clinical roles,^6^ this presents a promising opportunity to engage them in, and facilitate their transition to, academic roles within HPE to support the expansion of health care professions workforces. Though non‐practising health care professionals are often integral to the delivery of high‐quality education, and represent a potentially increasing demographic of educational faculty, emerging evidence suggests non‐practising professionals face significant career challenges in academia, including within HPE.

Indeed, the transition from bedside to academia poses distinct challenges. This begins with unclear entry paths into academia and is compounded by job demands. Challenges can lead to a sense of lost identity; the latter being especially pronounced for individuals with minoritised identities.^4^, ^7^, ^8^ Although a number of publications speak to, or around, this issue strictly within the context of *individual* health professions,^2^, ^4^, ^5^ there are no existing reviews that have synthesised evidence *across* professional and geographical lines. Given the importance of different contexts across the HPE disciplines, gathering this breadth of data would allow meaningful comparisons to be drawn and thus good practice to be shared and replicated where applicable. This sharing of ideas encourages a more comprehensive understanding of the experiences that non‐practising health care professionals face in educational settings and how they may best be supported in these roles.

In response, we present a scoping review exploring: ‘What is known about the career experiences of non‐practicing healthcare professionals (defined as individuals with clinical backgrounds who no longer engage in direct patient care) across various professions and internationally, within the field of health professions education?’. Whilst we acknowledge that those who leave clinical practice for the education sector might be involved in a multitude of different roles, leadership, administration and research, our scoping review is firmly focused on those directly involved in education. The reason for this is that educators have an, arguably, more direct impact on the training of our future health care workforce. They therefore play an active role in the response to the current and future demands to increase the workforce across the health professions.

The scoping review methodology aligned with our aim to map the content and quantify the scope of the existing literature on our topic of interest. Our broad research question and anticipated paucity of information on the topic required a wide net to capture all relevant articles, well‐suited to the scoping review approach, which favours a broad spectrum of article types (Thomas et al, 2020).^9^, ^10^ A scoping review provides ‘a clear indication of the volume of literature and studies available as well as an overview (broad or detailed) of its focus’^11^ and is better suited to broader research questions than systematic reviews.^12^ Hence, we selected this methodology to capture an interprofessional perspective on a non‐specified range non‐practising health care professionals' experiences within educational roles. Finally, Tricco et al.^13^ state that scoping reviews are often used to identify gaps for future initiatives. This aligns with our aim to inform practice by providing a foundation for recruitment and retention strategies for these highly skilled educators and direct future research into potential solutions to workforce challenges.

For the purposes of disseminating the results of this scoping review, we required a phrase that accurately represented the heterogenous group of different terms for health care professionals who no longer practise clinically and now devote their time to educational delivery within the HPE sector. Although there are many phrases in use, some of which have been identified as potentially negative (i.e. ‘non‐clinical’ being a phrase that only exists in opposition to ‘clinical’ and therefore arguably being devalued) or ambiguous (e.g. ‘academic healthcare practitioner’ that could encompass any scholarly, research or leadership role within HPE),^5^ we have chosen to use the term ‘clinically trained health professions educationalist’ or ‘CTHPE’ from this point forwards.

## 2 METHODS

Prior to conducting this scoping review, a protocol was devised and published^14^ and has been registered with the Open Science Framework (ID: https://doi.org/10.17605/OSF.IO/485Z3).

The conduct of the scoping review adhered to Arksey and O′Malley's^15^ guidelines:

Stage 1: Identifying the research question

The research question of this scoping review was ‘What is known about the career experiences of non‐practicing healthcare professionals (where non‐practicing is defined as individuals with clinical backgrounds who no longer have clinical roles directly relating to patient care) across professions, and internationally, in health professions education?’. There were no restrictions on article‐type (empirical research articles, editorials, reports and opinion pieces) or year of publication.

Stage 2: Identifying relevant studies

Seven literature databases were searched specialising in medicine, psychology and social science (Medline, Allied and Complementary Medicine [AMED], EMBASE, Psych INFO, CINAHL, ERIC and Scopus). Additional grey literature including research dissertations/theses were identified by searching ProQuest dissertations and theses A&I and OpenGrey archive databases. Google Scholar was also searched to capture any additional articles not identified by the more formal search tools. Searches, including database selection and search terms, were guided by a university librarian and were completed between 9th March 2023 and 11th April 2023.

Appendix A details all search terms used for each database. The search terms aimed to be consistent across databases, but formats were accordingly tailored to individual databases where necessary.

Forward and backward citation searching was also undertaken; backward citation was performed on all included articles discovered from database searching by reviewing the titles of article references for eligibility. Forward citation was performed on 7 key papers identified by the reviewers that were considered optimally aligned with our research question. Each of these papers was entered into Google Scholar and their citations screened for eligibility.

Stage 3: Study selection

All articles discovered through database/citation searching were considered regardless of article type or quality. Articles not accessible in the English language were excluded to avoid translation error.

**FIGURE 1:**
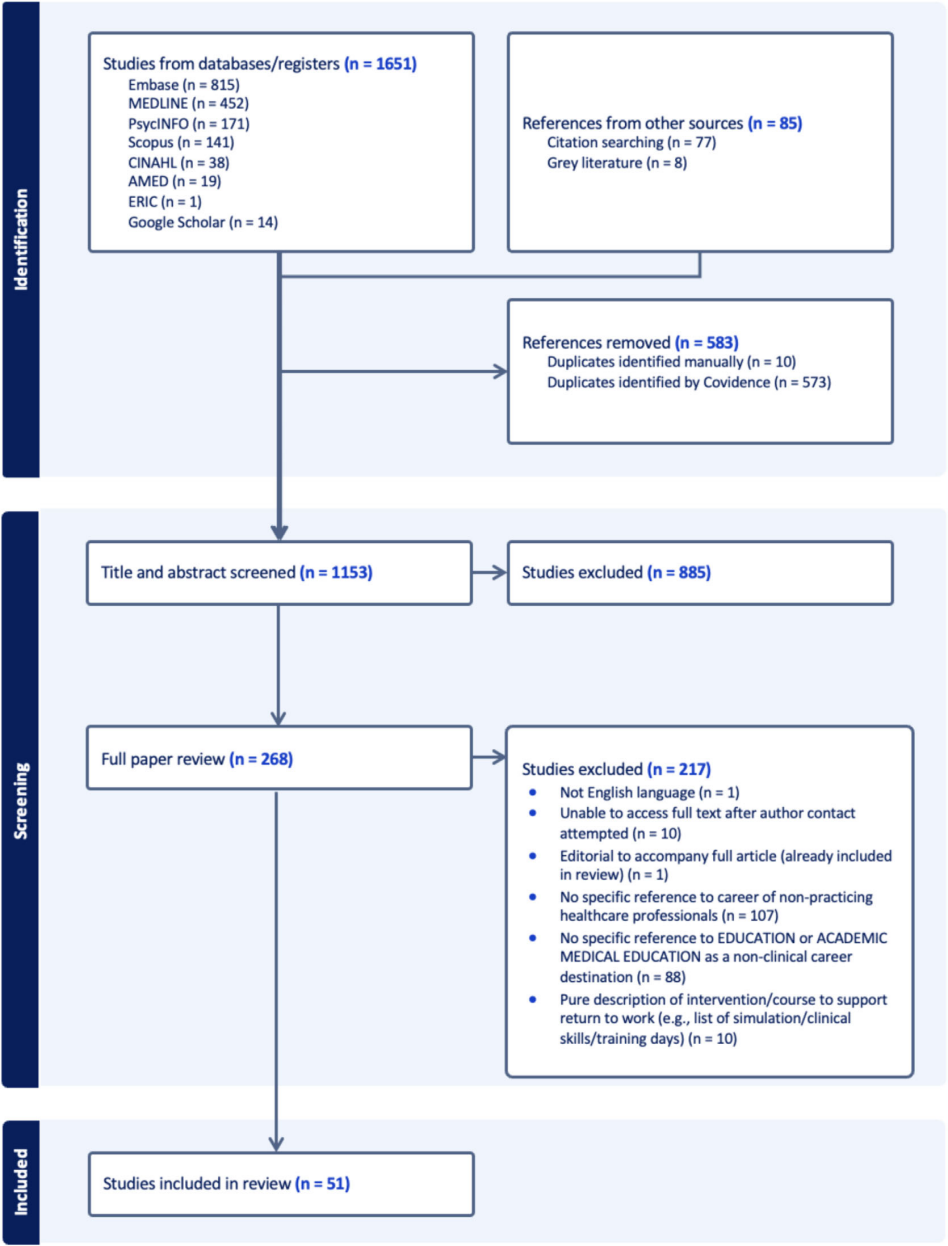
PRISMA diagram for the scoping review. [Color figure can be viewed at wileyonlinelibrary.com]

Each article identified was subjected to sequential title and abstract screening (undertaken by two reviewers) and full paper screening (single reviewer) to achieve final inclusion to the review. Figure 1 demonstrates this process through the PRISMA flow diagram.^16^

Stage 4: Charting the data

Initial data charting was completed using Covidence software for both the quantitative and qualitative aspects of this mixed‐methods analysis. An online extraction template (Appendix B) was created and applied to each included article to capture both the ‘demographic’ attributes of each article (e.g. date of publication and style of article) and any data (primary codes) related to the predefined, deductive qualitative topics.

Stage 5: Collating, summarising and reporting the results

Quantitative data pertaining to article attributes were downloaded and analysed using descriptive statistics. Regarding qualitative data, primary codes were entered into a spreadsheet, and each overarching topic was explored across the contributing articles independently by two reviewers to refine codes and themes until a thematic model was developed.

## 3 RESULTS

Of the 1736 articles originally identified through database searches (1651) and other sources (n = 85), and following removal of duplicates (n = 583), 1153 articles underwent title and abstract screening. A further 885 articles were excluded at this stage; therefore, 268 articles underwent full paper review; 217 articles were deemed not suitable for the review (for exclusion criteria breakdown, see Figure 1), leaving 51 eligible articles for analysis on the topic of the experiences of non‐practising clinicians who had made the transition to become a CTHPE within HPE. In this results section, we present the data through both a quantitative summary of article demographics (article type, publication date, country of origin and empirical data, where applicable) and a thematic analysis. In doing so, we map the existing literature in terms of volume, distribution and nature.

### 3.1 Quantitative analysis

Table 1 contains a summary of all quantitative data. Articles' first authors were predominantly based in the USA (n = 26), with other countries making more minor contributions to the literature (UK n = 8; Australia n = 7; Canada n = 2). Countries with a single contribution to the review are New Zealand, Ireland, South Africa, Spain, the Netherlands, Malta, Thailand and a collaboration between the UK and Australia. There has been a clear increase in the number of articles published on the topic from the 1990s (n = 4) to the 2010s (n = 25; average annual publication rate = 2.5 articles/year). In the 2020s, so far, there have been 8 articles published, suggesting a slight increase in interest, with an annual publication rate of 2.67 articles/year.

**TABLE 1:** Characteristics of included articles.

Article characteristic	Variable	Number of articles
Country in which the study conducted		
	United States	26
	UK	8
	Australia	7
	Canada	2
	Ireland	1
	Malta	1
	Netherlands	1
	New Zealand	1
	South Africa	1
	Spain	1
	Thailand	1
	Australia and UK collaborative	1
Year of publication		
	1990–1999	4
	2000–2009	14
	2010–2019	25
	2020–2023	8
Type of article		
	Commentary	17
	Research Paper—qualitative	16
	Research Paper—quantitative	8
	Research Paper—mixed methods	1
	Thesis/dissertation	4
	Literature Review	3
	Editorial	1
	Personal reflective account	1
Health care professional group (subject of article)		
	Nurses	33
	Doctors	6
	Occupational therapists	3
	Midwives and nurses	1
	Midwives	1
	Chiropractor	1
	Physician assistants	1
	Pharmacists	1
	Physiotherapists	1
	Multidisciplinary: nurses, occupational therapists, speech language pathologists	1
	Multidisciplinary: nursing and allied health (occupational therapists, physiotherapists, speech pathologists, dietitians, podiatrists)	1
	Unclear cohort: mixture of ‘clinician‐educator’ and ‘educator and previous clinicians’	1
Number of participants (Research Papers only)		
	0–9	8
	10–19	8
	20–29	1
	30–39	1
	40–49	0
	50–99	1
	100–149	2
	150–199	3
	200–249	0
	250–299	1
	300+	1
	Not clear/not stated	4

Commentaries were the most common article type (n = 17), closely followed by qualitative research articles (n = 16), and then quantitative research articles (n = 8). In the empirical research included, the number of participants ranged from 3 to 541. By far the most common professional group represented in our data is nursing, with 33 of all 51 articles focusing solely on nurses' experiences. In contrast, other professional groups represent only pockets of interest: doctors (n = 6); occupational therapists (n = 3); midwives (n = 2; one in combination with nurses); physiotherapists (n = 1); pharmacists (n = 1); and so on. The research articles generally reported small cohorts of participants, typically less than 20 participants (n = 16), with a further 9 studies reporting between 20 and 300 participants. The largest study included 541 participants. Appendix C contains the demographics of each included article.

### 3.2 Qualitative, thematic analysis

**FIGURE 2:**
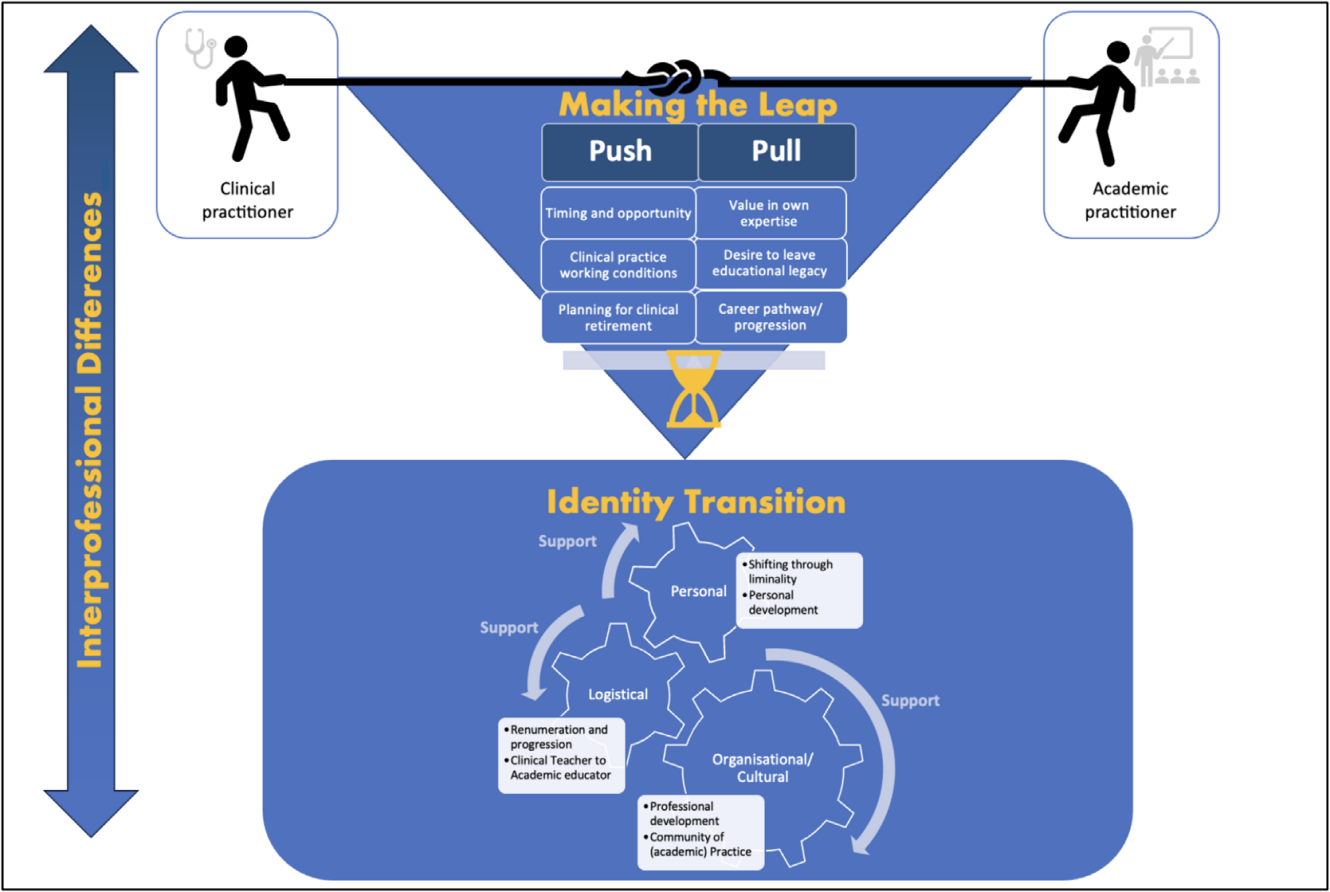
Thematic diagram of qualitative analysis showing the three major themes of ‘making the leap’, ‘identity transition’ and ‘interprofessional differences’. [Color figure can be viewed at wileyonlinelibrary.com]

The journey from clinical or clinical‐academic employment to solely academic work can be complex and challenging. Our studies suggest that the transition into academic employment is more than just a simple professional change; it is a complete transformation that includes financial, emotional, personal and vocational dimensions. The results of our thematic analysis are presented initially as exploring the uniting experiences across all professional disciplines of ‘making the leap’ and ‘identify transition’, before then considering the nuances of ‘inter‐disciplinary differences’ within this journey. The thematic diagram (Figure 2) shows how these themes are linked.

### 3.3 Making the leap

Within the wider literature, the term ‘making a leap’ has been used to describe the experiences of a heterogeneous group of health care practitioners (including clinical, non‐practising or scientific) transitioning to become CTHPEs. Similarly, this phrase captured the experiences of the non‐practising subjects within our review. Whilst the phrase encompasses the physical move from one professional career and environment to another, it perhaps more significantly represents the many different psychological, mental and identity‐based transformations that occur during this transition. It also emphasises the enormity of this transformation^7^, ^17^, ^18^ and the adaptability (and perhaps bravery) required of these individuals.^19^, ^20^ These practitioners simultaneously encounter the challenges of navigating new academic duties whilst struggling with the changing nature of their professional identity.^21^, ^22^, ^23^ These tensions between clinical and academic career pathways are demonstrated as the ‘tug of war’ in the thematic diagram (Figure 2).

#### 3.3.1 Timing the leap

It is important to note that not all professionals chose to make a sudden leap to the HPE sector, for some it was a more *gradual* transition, represented in the thematic diagram (Figure 2) as a sand timer. For example, Grabanski^24^ reports in an interview study that 10 out of 11 occupational therapists who had made the transition to academia kept ‘a foot in the clinic’ when starting out in academia to ease the transition; Lee et al.^25^ note the desire of those making the transition to academia to maintain connections to their clinical identities early during transition, and Neese^20^ describes the transition as most likely to be ‘protracted and cumulative’.

Most of the quantitative‐style research articles included in this study attempted to calculate the prevalence of those leaving the health professions entirely for educational roles or the frequency of reasons cited for leaving. Unfortunately, however, as a collective, they did so in heterogeneous ways that defy simple meta‐synthesis; in most instances, HPE as a career destination is reported incidentally or not the primary focus of the study. For example, in their study of why trainees choose to leave hospital‐based speciality training, Bustraan et al.^26^ report 20 out of 174 (11.6%) surveyed trainees continuing their career in a non‐clinical role, with ‘non‐clinical medical educator’ and ‘researcher’ both reported in non‐clinical career destinations but with no further breakdown of data. Duffield et al.^27^ report that 16% of 154 nurses leaving clinical practice in Australia enter ‘educational’ positions, though ‘education’ is broadly representative of health, school education and finance. These studies demonstrate that, across included studies, there is a lack of focus and standardisation on reporting the prevalence of those leaving practice for medical education.

#### 3.3.2 Push factors

The reasons practitioners choose to leave for HPE as viewed through the more qualitative research lens yields richer findings; we conceive of *push* factors as those reasons that make people want to leave clinical practice for HPE and *pull* factors as those that attract people to HPE from clinical practice. Several studies focused on the decision‐making factors influencing practitioners who had made this leap, encouraging them to reflect on their past experiences and suggesting that the decision to leave clinical practice is a key decision point, representing the beginning of practitioners' new careers within HPE.

Working conditions were a significant push factor that drove many practitioners, across career stages, away from clinical practice. Poor staffing levels, heavy workload (including heavy physical workload), insufficient resources, negative organisational culture and the continual pressure to satisfy administrative and patient care expectations often led practitioners to pursue alternative careers in HPE (Axiak and Axiak, 2024).^28^, ^29^, ^30^, ^32^, ^34^ In some cases, this led to burnout, which became a driving factor in making the leap.^29^ For more senior practitioners, the nearing or advent of retirement also raised practical concerns about ongoing job security, acting as a push factor to transition to an academic role that would offer longer‐term stability of employment, without the pressure of clinical work.^33^, ^35^ New mothers were noted in one study as experiencing a similar push (Marciano, 2013).^3^ Several studies in our review describe being in the right place, at the right time in terms of accessing opportunities within HPE.^17^, ^23^, ^33^ Though not entirely a push factor, an attractive opportunity in academia ‘being dropped in their lap’^33^ was highlighted as a factor influencing *who* gets to make the leap to HPE, as was the availability of the personal means and support to ‘take a step into the unknown’.^29^

#### 3.3.3 Pull factors

On the contrary, the appeal of working within HPE manifested in a variety of ways. Many individuals felt that their clinical expertise would be recognised and valued, believing they possessed a wealth of knowledge, and experiences that could significantly contribute to the development of a future generation of clinicians (Axiak and Axiak, 2022).^23^, ^34^, ^36^ Building on this, many practitioners seemed keen to leave a legacy in education that would positively influence patient care.^24^, ^37^, ^38^ For many, the promise of career progression within academia (particularly within nursing, where significant faculty shortages offered promises of promotion^38^, ^39^, ^40^) was appealing. Some studies report the appeal of clear career pathways that are perceived as rewarding excellence (particularly the tenure pathway within the USA),^33^, ^41^ but international differences exist—for example, the UK was noted to have a lack of a clear career path for practitioners making the transition to HPE.^7^

### 3.4 Identity transition

Once practitioners have made the leap to working only within medical education roles, and begin to settle into their new working lives, they experience significant shifts in their professional identities^22^ that commonly lead to feelings of isolation, embarrassment,^21^ alienation,^25^, ^42^ judgement,^18^ frustration,^43^ demotivation,^25^ fear,^23^ grief and loss.^25^, ^44^, ^45^ Participants repeatedly expressed the serious, and often challenging, emotional and psychological changes that accompanied their transition,^25^, ^46^, ^47^ and some of these were associated with difficult feelings of ‘starting over’,^48^ or perceptions that specialised clinical skills were no longer valued.^25^ Support from colleagues and organisations greased these ‘cogs’ of identity transition, as demonstrated in Figure 2, and the presence of such support should not be underestimated.

#### 3.4.1 Shifting through liminality

Participants described the sensation of feeling as though they were ‘living in two worlds’,^49^ oscillating between identities,^7^ in a continuous state of ‘newness’,^48^ transitioning between social statuses,^17^ ontologically insecure^18^ or existing in a liminal state.^36^, ^50^ These changes were often in response to periods of difficulty or barriers faced within their academic practice.^47^ Cumulatively, and over time (some studies tried to define this time period, with more than one study suggesting transitioning to an academic role takes up to 3 years^22^, ^33^, ^51^), participants' new roles led to shifts in their own perceptions of their professional identity (Grassley, 2020).^37^, ^51^ Several studies noted a return to confidence or ‘rebirth’ for educators following, and resolution of, experiences of challenges related to identity development.^25^, ^44^, ^49^ There did not seem to be consistency in relation to the labelling of this evolved identity, with Lee et al.^25^ suggesting some practitioners' primary identities firmly become educational, whereas others remain firmly clinical, even if they no longer practice.

#### 3.4.2 Remuneration and progression

A stark difference in salary was one of the first difficulties highlighted in this review. Several studies expressed worries about the disparity in pay,^25^, ^29^, ^39^, ^52^ with higher rates of pay for clinical work leading to not only personal difficulties and as a barrier for engagement (for example,^52^ reports that average salaries for full‐time nursing faculty are equivalent to entry‐level practising nurses) but a sense of resentment and bitterness developing towards practitioners' new role in medical education.^30^ Further, concerns about student loan debt compounded participants' financial concerns.^53^ Additionally, tenure and promotion processes were described as unpredictable and stressful, and understanding how these processes function added to the stress practitioners during the transition to a new role.^55^

#### 3.4.3 Clinical teacher to academic educator

Discovering that clinical knowledge, or even clinical education knowledge, does not always equip one for the complexity and rigour of academic teaching was a challenging realisation for many participants (Schriner, 2007).^19^, ^32^, ^33^, ^49^, ^77^ Both undergraduate and postgraduate education prepare students for clinical practice, or at best for clinical teaching practice, rather than for faculty roles,^20^, ^28^, ^56^ or alternative careers more broadly.^29^ Many openly admitted naively believing that years of clinical practice would naturally translate to academic competence^49^, ^50^ or that experience of clinical teaching would fully prepare them for a faculty role.^19^, ^33^ However, participants confessed that their reality was frequently different to their assumptions and found it difficult to ask for help, particularly if they were experienced clinicians more accustomed to providing answers than asking for them.^37^, ^43^, ^49^ With varied and diverse teaching approaches, dependence on educational theory, new operational structures including academic ranks and new terminology, participants transitioning to academic HPE experienced a steep learning curve,^43^, ^57^, ^58^, ^59^ described by some as learning the ‘secret handshake’ of academia.^49^ McDermid et al.^19^ highlight how part of this learning involved coming to realise that a PhD was essentially currency for progression of non‐practising staff within academia. Where participants admitted to underestimating the complexities of HPE, they often experienced feelings of inadequacy and self‐doubt,^43^ which could lead to strategies involving over‐compensation, and over‐preparation, placing newly transitioned staff at high risk of burnout.^19^, ^32^ Neese (2003)^20^ highlights how critical self‐reflection, including questioning and examining one's assumptions related to academia, is key to successfully navigating this.

#### 3.4.4 Personal and professional development

Though shifts in identity coupled with organisational barriers created challenges, there were positive aspects related to identity development and growth reported by included studies. Initially, new educators showcased high levels of optimism and enthusiasm for HPE, with many keen to influence the educational environment using their unique perspective of practice. Though initial optimism waned for some, it was maintained for others, and many found HPE to be a rewarding career, particularly when they were able to assist students in mastering new skills or concepts^52^ or when autonomous practice was facilitated.^19^ Opportunities for professional development (e.g. collaboration and research) were reported, which encouraged comfort and confidence in one's role.^52^, ^60^ Other factors that validated new academics' sense of legitimacy included positive student feedback^49^; witnessing the impact of teaching in relation to student outcomes^32^; and developing an area of educational expertise perceived as ‘useful’ to an institution, for example, curriculum development.^7^

#### 3.4.5 Community of (academic) practice

Much of the literature we reviewed highlighted the importance of support for becoming a CTHPE in HPE, though this was noted as inconsistent,^32^, ^61^ particularly in relation to formal inductions.^40^, ^48^ Support in various forms—such as with recruitment,^28^ networking,^43^ interpersonal issues,^44^, ^47^, ^59^ finances and academic performance^48^—creates opportunities for new practitioners to thrive.

Critically, support should not just be for new practitioners' initial transition to their roles, as a period of ‘drowning’ is experienced when initial supervisory and support structures are removed.^49^ Some studies noted collegial support as most important to successfully transitioning to HPE,^38^, ^44^, ^48^, ^51^ as this can help build belonging.^18^ Interestingly, Kreger^43^ notes that the more connected a professional is to academia before they transition, the more successful their transition is, perhaps suggesting the power of a gradual shift to educational practice. When support is completely absent—for example, interpersonal support can be difficult to access due to lack of provision of formal mentors and staffs' busy academic workloads^42^—then practitioners can struggle to surmount barriers, adjust to shifts in their working environment, and identity,^7^, ^22^, ^45^ and can easily become burnt out.^23^

### 3.5 Interprofessional differences

Though our data are predominantly situated within the context of nursing, we noted important interprofessional differences in experiences, for example, between the experiences of nurses and doctors. Most notable is that most of our data within nursing focuses on the experience of becoming an academic—of transitioning to the world of academia, its norms, conventions and rules (e.g. previous studies^33^, ^43^, ^44^, ^48^, ^55^, ^61^). This contrasts with literature from medicine, where the more limited literature available focuses on the experience of those who leave clinical practice (i.e. become ‘non‐clinical’) (e.g., other works^7^, ^29^). Though the difference between becoming an academic and becoming non‐clinical may seem semantic, it is important and tells us something critical about the differences in norms between nursing and medicine. In nursing, transitioning to academia whilst maintaining a clinical licence is relatively common and so has become embedded in the field's traditions and norms, though we did note debate on this topic occurring during literature retrieved from the 1990s/early 2000s, with some strong advocates of the need for nurses in education to remain certified,^68^ and concerns regarding the lack of time to do so due to heavy teaching loads.^39^, ^59^

In contrast, the emphasis on becoming non‐clinical, rather than an academic, in doctor‐focused literature implies that there is something extraordinary about the decision not to practice. The literature suggests significant stigma associated with stepping away from direct patient care within medicine—as Church and Brown^7^ describe, becoming non‐practising involves significant loss of cultural capital and can lead to external negative judgement from colleagues and institutions. Govender^29^ describes how stigma can be internalised into deep feelings of shame and fear in relation to leaving practice. Early‐career women leaving medicine are more likely to experience such barriers.^7^ The requirement in medicine that practitioners must be engaged in active clinical work to maintain their licence exacerbates this divide. Whilst nursing views academia as an extension of clinical practice, medicine perceives it as a departure. Literature relating to other professions did not suggest a focus either aligned with nursing or medicine's focus here, though the numbers of papers from outside of nursing and medicine in this study are small, and so it is not possible to comment definitively on the experience of other health professions.

## 4 DISCUSSION

This scoping review highlights the complex and often challenging experiences of health care professionals transitioning from clinical roles to academic positions within HPE. Here, we discuss how our findings relate to broader literature, such as licencing and developments in professional identity whilst showcasing the unique challenges and opportunities that lie beyond the bedside for non‐practising health care professionals moving into educational roles.

### 4.1 Licencing implications

The transition from clinical practice to academia for non‐practising professionals often involved challenging issues with clinical practice licencing. Maintaining a licence to practice is desired by many making this transition,^5^ but as licencing varies between international, and professional, contexts, navigating this transition and/or managing licencing requirements across professions and geographies becomes increasingly complex. The journey to identifying as a non‐practising professional is rarely linear—often along the way, professionals will maintain some clinical responsibilities on a less‐than‐full‐time or locum basis, reducing clinical commitments more gradually over time. Perceptions of competency and ideas relating to professional identity also appear entwined with licencing, with many viewing a licence to practice as a symbol of professional competence, even for those not actively practising in clinical roles.^5^ This leads to many educational job descriptions requiring a licence to practice.

The requirement to maintain a licence becomes problematic for individuals choosing to become non‐practising. Particularly within nursing in the United States (USA), transitioning professionals face significant barriers (for example, finding adequate time) to maintaining licensure alongside fulfilling academic roles (Logan 2016).^39^, ^59^, ^61^ Within the UK, the General Medical Council (GMC, n.d.) does not recognise medical education as a specialty for which a doctor requires a licence to practice and therefore does not provide a pathway to revalidation through educational practice. However, conversely within the UK, nursing registration *can* be maintained in full‐time educational roles (Nursing and Midwifery Council, n.d.). Such variability in licencing requirements across professions and countries underscores the need for a more coherent and supportive approach to professional licencing. One suggestion would be to recognise HPE as a speciality deserving of its own licensure, ensuring HPE practitioners held both clinical and educational credentials (as opposed to the current trend in many countries where clinical currency overpowers educational expertise^7^). This could ease the transition for non‐practising professionals and increase the perceived legitimacy of this career path.

### 4.2 Navigating identity liminality

Central to our findings is the transition in professional identity experienced by clinicians moving into academic roles, framed using the concept of liminality. Two papers make a connection explicitly to liminality,^36^, ^50^ whereas others, which use phrases like ‘living in two worlds’,^49^ oscillating between identities^7^ and ontological insecurity,^18^ suggest a liminal character to this transition in identity.

Liminality is characterised by a sense of being ‘betwixt and between’ (van Gennep, 1960)^62^ traditional professional identities in this review (i.e. the identity of doctor, nurse and occupational therapist; and the identity of educator, academic, researcher, leader, etc.) and represents a significant challenge for non‐practising professionals. Indeed, van Gennep (1960),^62^ the progenitor of liminality as a concept, describes the process as ‘existential’, highlighting the profound impact this transition can have on an individual's sense of self and belonging.

Liminality is increasingly discussed as playing a role in several identity transitions within HPE, including from trainee to trainer (Gordon, Rees, and Jindal Snape, 2020),^63^ from medical student to doctor (Brown et al 2021)^65^ and within training programmes at periods of transition in responsibility (Kerins, Smith and Tallentire, 2023)^64^ but has not been discussed in relation to the experiences of non‐practising professionals in HPE. What this review suggests is that we need to broaden our understanding of, and focus on, liminality within HPE—it is not restricted to conventional progression pathways, and extends to, and may disproportionately negatively affect those, transitioning from clinical practice to academic roles.

Liminality involves ‘boundary work’ or active navigation of shifts in one's professional self‐concept that can be existentially challenging (Paulson and Hernes, 2003).^66^ This challenge is emphasised where support for guidance through liminality is lacking, where there is no clear progression out of liminality (i.e. a next step in a role hierarchy) and where cultural narratives do not support the legitimacy of the person experiencing liminality (Ibarra and Obadaru, 2016).^67^ As non‐practising professionals are often forging their own career paths with high levels of instability and uncertainty, and may face stigma and loss of status, the negative impacts of liminality, therefore, may be more likely. There is a lack of research regarding non‐practising professionals' experiences of liminality—an important direction for future research, given the indication of liminality within our data, its close association with identity development, and the unknown, and possibly negative, impacts on non‐practising clinicians.

### 4.3 Critical need for support

Many of the articles in this scoping review (Axiak and Axiak, 2022)^28^, ^29^, ^30^, ^32^, ^34^ suggest that educational practice may be sought as a viable alternative to the stressors clinical practice. However, they simultaneously underscore that educational practice, whilst offering an alternative to clinical practice, brings its own set of challenges. These include administrative burdens, time pressures and the added responsibilities of research and publication.^43^, ^57^, ^58^, ^59^ Acknowledging these challenges is crucial in developing recruitment and retention strategies that are tailored to support health care practitioners making the leap to academia. Ultimately, jobseekers with a clinical background should be encouraged to realistically examine educational practice; seeing it merely as an ‘escape route’ may make jobseekers less aware of the potential (significant) challenges that await in academia.^69^

In making the leap to education, there ensues a period of identity renegotiation that also demands institutional support. Indeed, our data^44^, ^47^, ^48^, ^59^ suggest that providing support helps new education practitioners succeed in their role. This is corroborated in the literature where structured induction and onboarding processes are recommended for new hires in HPE.^70^, ^71^, ^72^, ^73^, ^74^ Daw et al.^75^ support this from an economics perspective, suggesting that a financial investment in new faculty has a promising return on investment and may be linked to improved retention of faculty. Acknowledging that an abrupt withdrawal of support is ill‐advised,^49^ long‐term mentoring relationships may be an ideal vehicle to retain and support new faculty.^76^

### 4.4 Working towards a diverse faculty

Whilst this review focussed on those who wished to leave clinical practice to pursue a career wholly dedicated to HPE, it is important to acknowledge that this potentially diverts human resources away from clinical practice and patient‐facing services. In contrast, the balance of a ‘clinical academic’ job satisfies both the service and education provision balance, and for many health professionals, pursuing an educational role is a widely used career route. However, for those who no longer wish to practice clinically, their primary health professions degree and any additional postgraduate training remain invaluable resources within a HPE setting, and their knowledge, skills and enthusiasm for education can be redirected into the training of future clinicians. The continuity of educational delivery is also an important factor that those no longer in clinical practice can deliver with more certainty; without the competing (and often dominant) pull of clinical work that must sometimes take priority over education and research delivery, full‐time educationalists have more time to embed themselves within the faculty and are perhaps equally, if not better placed, to deliver day‐to‐day overarching leadership and management roles.

Of course, clinical academics may be well placed to deliver specialty‐specific teaching within any HPE department, but in addition to clinical knowledge and skills, a key objective of HPE is to create self‐sufficient, adult learners who have the necessary skills to continue their own professional development postgraduation.^54^ This requires acquiring non‐clinical, academic skills to future‐proof ones own career trajectory, which could equally be delivered and nurtured by clinical or non‐clinical staff.

In summary, it takes a village of people to train the future health care workforce, especially in the light of current and future health care workforce targets. Therefore, considering diversity in hiring and progression pathways pertaining to current practice, previous experience and additional academic skills (e.g. higher degrees and educational qualifications) can ensure that talented and capable individuals are not being overlooked for educational roles based on ideas of clinical currency.^7^

### 4.5 Limitations

Despite steps taken to ensure a rigorous and thorough scoping review, it is limited by the lack of diversity of available data. Most papers relate to nursing, which limits transferability of findings to other health care professions (e.g. medicine). This could be attributed to historical recruitment patterns and gender disparities or disparities in professional rewards across backgrounds. The geographical diversity of papers in the review was also limited with the vast majority from one of five countries (UK, USA, Canada, Australia or The Netherlands). This may represent a language bias within the study as only articles in English were reviewed to avoid mistranslation. Future work should, where possible, broaden the scope of inclusion criteria to enhance the diversity and representativeness of the findings.

### 4.6 Conclusion

This scoping review highlights the varied and significant challenges faced by health care professionals transitioning from clinical roles to educational positions within HPE. From navigating licencing complexities to grappling with liminal identities, the journey from bedside to lecture theatre is often challenging. Our findings showcase the importance of recognising the challenges liminality poses to individuals undergoing this transition, as they navigate the blurred boundaries between traditional professional identities. Further, our review calls for a more coherent and supportive approach to both professional licencing across contexts and to institutional support for new hires.

This synthesis also highlights absences in the literature and subsequent areas for future research. In general, there is a relative paucity of evidence regarding the roles and experiences of non‐practising professionals in HPE, particularly for professionals from non‐nursing backgrounds. In future, adopting an intersectional approach to exploring these professionals' experiences would be beneficial in developing a more nuanced understanding of this demographic, in particular around the conceptualisation of becoming ‘academic’ or ‘non‐clinical’ within different professions, and how these may be challenged to allow individuals to thrive within educational spaces. In addition, the results of this review are heavily weighted towards the themes around transitioning to the role of CTHPE from a role in active clinical practice. This was an unanticipated, albeit interesting, outcome of the review. Increased numbers of publication on this transition may indicate increased interest in this transition and/or early years following this career move. However, it may also indicate issues with more established CTHPEs—either their perspectives have not been solicited, or there is a leaky pipeline leading to loss of more established educators and their perspectives. Regardless, given the increasing exodus from clinical practice, we are cautiously optimistic that the body of evidence pertaining to more established CTHPE positions will feature more prominently in literature in the years to come, encouraging the development of rich research in this area.

In the meanwhile, acting on the findings of this review, we can begin to create more inclusive and supportive environments in HPE for faculty. We recommend that educational institutions recognise and actively engage with this increasing demographic of health care professions educationalists to address academic faculty shortages and empower individuals to make meaningful contributions to HPE and the demand for health care workforce expansion. More specifically, providing more support for the clinician transferring to a full time education role is key. For those governing clinical licensure across different disciplines worldwide, consideration given to the prospect of maintaining a clinical licence through purely educational roles would go a long way to reocgnise this group of clinicians in education as distinct from, but whose role in training the future workforce is just as important as, clinical academics.

## AUTHOR CONTRIBUTIONS

**Helen R. Church:** Conceptualization; project administration; data curation; methodology; writing—original draft; software; visualization; formal analysis; supervision. **Megan E. L. Brown:** Conceptualization; writing—original draft; writing—review and editing; formal analysis; data curation; project administration; methodology. **Lynelle Govender:** Conceptualization; methodology; formal analysis; project administration; data curation; writing—review and editing; writing—original draft. **Deborah Clark:** Conceptualization; writing—original draft; methodology; writing—review and editing; formal analysis; project administration; data curation.

## CONFLICT OF INTEREST STATEMENT

The authors declare no conflict of interest.

## ETHICS STATEMENT

Ethical approval was not necessary for this scoping review.

## Data Availability

The data that support the findings of this study are available in the supplementary material of this article.

## APPENDIX A

**Table:**

Database	Full search strategy
Medline	exp Health Personnel/OR (doctor* or nurse$ or clinician* or chiropodist* or podiatrist* or dietician* or orthoptist* or radiographer* or paramedic* or physiotherapist* or osteopath* or pharmac* or optician* or chiropractor* or radiographer* or “language therapist*” or “speech therapist*” or pyschologist* or prosthetist* or “occupational therapist*” or “operating department practitioner*]” or “arts therapist*” or “biomedical scientist*” or “clinical scientist*”).mp. OR (health* adj3 (worker* or provider* or professional* or personnel)).mp. AND exp Education, Medical/OR exp Health Education/OR exp Vocational Education/OR exp Education, Professional/OR exp Education/OR exp Teaching/OR exp Curriculum/OR (educat* or teach*).mp. AND (“non practic*” or “ex practitioner*” or “no longer practic*” or “non practis*” or “no longer practis*” or “ex clinician*” or “non clinical role*”).mp. OR (non adj2 (practic* or practis*)).mp. OR (ex adj2 practitioner*).mp. OR (career adj2 change).mp. [mp=abstract, heading words, title] OR (career adj2 transition).mp. [mp=abstract, heading words, title] OR career change.mp. OR career transition.mp. OR
AMED	
EMBASE	
PyschINFO	
CINAHL	““(doctors or physicians or healthcare professionals or nurses) AND (education or school or learning or teaching or classroom or education system) AND ((“non practic*” or “ex practitioner*” or “no longer practic*” or “non practis*” or “no longer practis*” or “ex clinician*” or “non clinical role*”))”“
ERIC	““(doctors or physicians or healthcare professionals or nurses) AND (education or school or learning or teaching or classroom or education system) AND ((“non practic*” or “ex practitioner*” or “no longer practic*” or “non practis*” or “no longer practis*” or “ex clinician*” or “non clinical role*”))”“
PEDRO	CLINICAL DATABASE—search engine not helpful for our topic https://search.pedro.org.au/advanced‐search
Scopus	(TITLE‐ABS‐KEY (doctor* OR nurse$ OR clinician* OR chiropodist* OR podiatrist* OR dietician* OR orthoptist* OR radiographer* OR paramedic* OR physiotherapist* OR osteopath* OR pharmac* OR optician* OR chiropractor* OR radiographer* OR “language therapist*” OR “speech therapist*” OR pyschologist* OR prosthetist* OR “occupational therapist*” OR “operating department practitioner*” OR “arts therapist*” OR “biomedical scientist*” OR “clinical scientist*”) AND TITLE‐ABS‐KEY (“education” OR “medical education” OR “medical educator*”) AND TITLE‐ABS‐KEY (“non practic*” OR “ex practitioner*” OR “no longer practic*” OR “non practis*” OR “no longer practis*” OR “ex clinician*” OR “non clinical role*” OR “career change” OR “career transition”))
Google scholar	healthcare AND education AND “non practic *” OR “ex practitioner *” OR “no longer practic *”
ProQuest Dissertations & Theses A&I	““(doctors or physicians or healthcare professionals or nurses) AND (education or school or learning or teaching or classroom or education system) AND ((“non practic*” or “ex practitioner*” or “no longer practic*” or “non practis*” or “no longer practis*” or “ex clinician*” or “non clinical role*”))”“
opengrey	““(doctors or physicians or healthcare professionals or nurses) AND (education or school or learning or teaching or classroom or education system) AND ((“non practic*” or “ex practitioner*” or “no longer practic*” or “non practis*” or “no longer practis*” or “ex clinician*” or “non clinical role*”))”“

## Appendix B EXTRACTION TEMPLATE

General information

**Title**

Title of paper / abstract / report that data are extracted from

**Lead author (surname, firstname)**

**Year of publication**

**Country in which the study conducted**

1. Australia
2. Canada
3. New Zealand
4. United States
5. UK
6. Other

Characteristics of included studies

**Type of article**

1. Research Paper ‐ quantitative
2. Research Paper ‐ qualitative
3. Research Paper ‐ mixed methods
4. Literature review
5. Commentary
6. Letter to editor
7. Thesis/dissertation
8. Abstract (e.g., for conference)
9. Other

**If research study…**

**Methods**

1. NOT RESEARCH STUDY
2. Questionnaire/Survey
3. Interviews (semistructured/structured)
4. Focus Groups
5. Other

**Total number of participants**

Enter number of participants if research study ‐ if not research, enter ‘N/A’ here

**Health care Professional Group(s) being discussed in article**

1. Dentists
2. Doctors
3. Midwives
4. Nurses
5. Occupational therapists
6. Pharmacists
7. Physiotherapists
8. Psychologists
9. Other

Thematic/Topic Analysis

**Inclusion criteria that applies to article**

1. REASONS for LEAVING clinical practice
2. PREVALENCE of LEAVING clinical practice (e.g., by specialty, gender, background, profession…)
3. Non‐practising experience/journey/job
4. REASONS for non‐practising health care professionals RETURNING to clinical practice
5. PREVALENCE of RETURNING to clinical practice (e.g. by specialty, gender, background, profession…)
6. Other

**Thematic Topics**

**Table:**

	**Text/codes from article supporting topic**
**REASONS for LEAVING clinical practice**	
**PREVALENCE of LEAVING clinical practice (e.g. by specialty, gender, background, profession…)**	
**Non‐practising job/role**	
**Non‐practising job location/geography (e.g. hospital, university, community…)**	
**Non‐practising experience ‐ positives**	
**Non‐practising job/role ‐ negatives**	
**Non‐practising job/role ‐ neutrals**	
**REASONS for non‐practising health care professionals RETURNING to clinical practice**	
**PREVALENCE of RETURNING to clinical practice (e.g. by specialty, gender, background, profession…)**	
**Licencing/practice restrictions/permissions**	
**Other**	

**Have you checked the references for potential articles of interest?**

1. Yes

**Key paper? (for forward citation searching).**

Does this paper make a significant contribution to the scoping review?

1. Yes
2. No

## Appendix C DEMOGRAPHICS OF EACH ARTICLE

**Table:**

Lead author, Year	Title	Country in which the study conducted	Type of article	Health care professionals discussed in article	Total number of participants
Esper, 1995^47^	Facing transition‐nurse clinician to nurse educator	United States	Commentary	Nurses	N/A
Diekelmann, 2004^42^	New Pedagogies for Nursing: Experienced Practitioners as New Faculty: New Pedagogies and New Possibilities	United States	Research Paper ‐ qualitative	Nurses	Not stated
Culleiton and Shellenbarger 2007^52^	Transition of a Bedside Clinician to a Nurse Educator	United States	Commentary	Nurses	N/A
Neese, 2003^20^	A transformational journey from clinician to educator	United States	Commentary	Nurses	N/A
Kenny, 2004^80^	Negotiating socialisation: the journey of novice nurse academics into higher education	UK	Commentary	Nurses	N/A
Hurst, 2010^51^	Experiences of new physiotherapy lecturers making the shift from clinical practice into academia	UK	Research Paper ‐ qualitative	Physiotherapists	8
Fain, 2011^56^	Helping More Practitioners Become Academics	United States	Commentary	Occupational therapists	N/A
Grabanski, 2014^24^	The experiences of occupational therapy clinicians transitioning to the role of faculty member: Implications for faculty development	United States	Thesis/dissertation	Occupational therapists	11
McDonald, 2010^59^	Transitioning from clinical practice to nursing faculty: lessons learned	United States	Commentary	Nurses	N/A
Hoffman, 2019^48^	Transitional experiences: From clinical nurse to nurse faculty	United States	Research Paper ‐ qualitative	Nurses	15
Dempsey, 2007^44^	The experiences of Irish nurse lecturers role transition from clinician to educator	Ireland	Research Paper ‐ qualitative	Nurses	6
Chargualaf et al., 2017^33^	The transition from military nurse to nurse faculty: A descriptive study	United States	Research Paper ‐ qualitative	Nurses	13
Weidman, 2013^23^	The lived experience of the transition of the clinical nurse expert to the novice nurse educator	United States	Research Paper ‐ qualitative	Nurses	8
Duffy, 2013^45^	Nurse to educator? Academic roles and the formation of personal academic identities	UK	Research Paper ‐ qualitative	Nurses	14
Anderson, 2009^49^	The work‐role transition of expert clinician to novice academic educator	United States	Research Paper ‐ qualitative	Nurses	18
Siler and Kleiner, 2001^32^	Novice faculty: Encountering expectations in academia	United States	Research Paper ‐ qualitative	Nurses	12 (but only 6 included in this study, as it focuses on expectations of becoming faculty)
Schriner, 2007^77^	The influence of culture on clinical nurses transitioning into the faculty role	United States	Research Paper ‐ qualitative	Nurses	7
Murray 2014	The transition from clinician to academic in nursing and allied health: A qualitative meta‐synthesis	Australia	Literature review	Nursing and allied health (OT, physio, speech pathology, dietetics, podiatry)	N/A (but 7 included studies)
Hunter and Hayter, 2019^36^	A neglected transition in nursing: The need to support the move from clinician to academic properly	UK	Editorial	Nurses	N/A
Grassley 2020^32^	No longer expert: A meta‐synthesis describing the transition from clinician to academic	United States	Systematic review	Nurses	N/A
McDermid 2013^16^	‚ÄòI thought I was just going to teach‚Äô: Stories of new nurse academics on transitioning from sessional teaching to continuing academic positions	Australia	Research Paper ‐ qualitative	Nurses	14
MacNeil, 1997^17^	From nurse to teacher: Recognising a status passage	UK	Research Paper ‐ qualitative	Nurses	Unknown. Author did not state
Logan 2016^55^	Transition from clinician to academic: An interview study of the experiences of UK and Australian registered nurses	Australia and UK	Research Paper ‐ qualitative	Nurses	14
Govender, 2023^29^	Separation anxiety: Walking away from clinical practice	South Africa	Commentary	Doctors	N/A
Choudhry, 1992^68^	Faculty practice competencies: nurse educators' perceptions.	United States	Research Paper ‐ quantitative	Nurses	291
McMurtrie et al., 2014^78^	Keeping our nursing and midwifery workforce: Factors that support non‐practising clinicians to return to practice.	Australia	Research Paper ‐ quantitative	Midwives; Nurses	158
Danna et al., 2010^60^	From practice to education: Perspectives from three nurse leaders.	United States	Commentary	Nurses	N/A
Marciano 2013^3^	Moving from clinical practice to academe: An analysis of career change for physician assistants.	United States	Research Paper ‐ quantitative	Physician Assistants	25
Kreger, 2019^43^	Down the rabbit hole: A grounded theory of health professionals' transitioning from practice to academia.	United States	Thesis/dissertation	Nurses, occupational therapists, speech language pathologists	8
Aguayo‐Gonzalez and Weise, 2022^46^	Career transition and identity development in academic nurses: A qualitative study.	Spain	Research Paper ‐ qualitative	Nurses	3
Richards 2017^33^	Towards late career transitioning: a proposal for academic surgeons	Canada	Commentary	Doctors	N/A
Duffield 2004^26^	Nursing: a stepping stone to future careers.	Australia	Research Paper ‐ quantitative	Nurses	154
Harms 2005^79^	A 25‐year single institution analysis of health, practice, and fate of general surgeons.	United States	Research Paper ‐ quantitative	Doctors	110
Beres, 2006^39^	Staff development to university faculty: reflections of a nurse educator.	United States	Commentary	Nurses	N/A
Schattner et al., 2007^31^	Impact of Master of Family Medicine degree by distance learning on general practitioners' career options.	Australia	Research Paper ‐ quantitative	Doctors	130
Jamieson and Taua, 2009^30^	Leaving from and returning to nursing practice: contributing factors.	New Zealand	Research Paper ‐ mixed methods	Nurses	32
Morrissette, 2011^55^	Recruitment and retention of Canadian undergraduate psychiatric nursing faculty: challenges and recommendations.	Canada	Commentary	Nurses	N/A
Mirtz et al., 2010^53^	Attitudes of non‐practicing chiropractors: a pilot survey concerning factors related to attrition.	United States	Research Paper ‐ quantitative	Chiropractor	70
Goodrich, 2014^41^	Transition to academic nurse educator: a survey exploring readiness, confidence, and locus of control.	United States	Thesis/dissertation	Nurses	541
Blackmer et al., 2018^57^	Clinical pharmacy academic career transitions: Viewpoints from the fieldPart 1: Understanding feedback, evaluation, and advancement.	United States	Commentary	Pharmacists	N/A
Bustraan 2019^23^	Why do trainees leave hospital‐based specialty training? A nationwide survey study investigating factors involved in attrition and subsequent career choices in the Netherlands.	Netherlands	Research Paper ‐ quantitative	Doctors	174
Church 2022^6^	Rise of the Med‐Ed‐ists: Achieving a critical mass of non‐practicing clinicians within medical education.	UK	Commentary	Doctors	N/A
Axiak 2022	An Exploration of the Transition of Clinical Nurses to an Academic Nurse Lecturer Role.	Malta	Research Paper ‐ qualitative	Nurses	8
Crist, 1999^28^	Career transition from clinician to academician: responsibilities and reflections	United States	Commentary	Occupational therapists	N/A
Ward, 2023^40^	Transition From Practice to Teaching: The Advice I Wish I Received	United States	Commentary	Nurses	N/A
Gould, 2016^50^	Metamorphosis: the journey from practice to education	UK	Commentary	Midwives	N/A
Seal, 2017^18^	Multiple professional identities: A personal exploration of the transition from nurse to lecturer	UK	Personal reflective account	Nurses	N/A
Fox, 2017^21^	The experience of nurse faculty new to a full time academic role and intent to stay in academia	United States	Thesis/dissertation	Nurses	14
Cleary et al., 2011^58^	Mental Health Nursing: Transitions From Practice Roles to Academia	Australia	Commentary	Nurses	N/A
Lee 2022^22^	Identities and roles through clinician‐educator transitions: A systematic narrative review	Australia	Literature review	Mixture of “clinician‐educator” (still practicing) and “educator + previous clinician” papers. (not clearly delineated)	N/A
Wongpimoln 2021	Transitional Experiences from Clinical Nurse Experts to Novice Nurse Lecturers in the University for Local Development in Thailand: A Phenomenological Study	Thailand	Research Paper ‐ qualitative	Nurses	8

## References

[1] WHO . Global Strategy on Human Resources for Health: Workforce 2030. WHO; 2016.

[2] Huwendiek S , Mennin S , Dern P , et al. Expertise, needs and challenges of medical educators: results of an international web survey. Med Teach. 2010;32(11):912‐918. doi:10.3109/0142159X.2010.497822 21039102

[3] Marciano GJ . Moving From Clinical Practice to Academe: An Analysis of Career Change for Physician Assistants (Doctoral dissertation,. Fordham University; 2013.

[4] Hu WC , Thistlethwaite JE , Weller J , Gallego G , Monteith J , McColl GJ . ‘It was serendipity’: a qualitative study of academic careers in medical education. Med Educ. 2015;49(11):1124‐1136. 26494065 10.1111/medu.12822

[5] Govender L , Church HR . When I say … ‘non‐clinical practice’. Med Educ. 2023;58(2):183‐184. 37517429 10.1111/medu.15177

[6] NHS England . (2023). NHS Long Term Workforce Plan. https://www.england.nhs.uk/wp-content/uploads/2023/06/nhs-long-term-workforce-plan-v1.2.pdf (Accessed 06/03/2024).

[7] Church H , Brown MEL . Rise of the Med‐Ed‐ists: achieving a critical mass of non‐practicing clinicians within medical education. Med Educ. 2022;56(12):1160‐1162. 36148497 10.1111/medu.14940 PMC9828655

[8] Varpio L , Harvey E , Jaarsma D , et al. Attaining full professor: women's and men's experiences in medical education. Med Educ. 2021;55(5):582‐594. 33034082 10.1111/medu.14392

[9] Thomas A , Lubarsky S , Varpio L , Durning SJ , Young ME . Scoping reviews in health professions education: challenges, considerations and lessons learned about epistemology and methodology. Adv Health Sci Educ. 2020;25(4):989‐1002. doi:10.1007/s10459-019-09932-2 31768787

[10] Sethi A , Ajjawi R , McAleer S , Schofield S . Exploring the tensions of being and becoming a medical educator. BMC Med Educ. 2017;17(1):62. doi:10.1186/s12909-017-0894-3 28335820 PMC5364693

[11] Munn Z , Peters MDJ , Stern C , Tufanaru C , McArthur A , Aromataris E . Systematic review or scoping review? Guidance for authors when choosing between a systematic or scoping review approach. BMC Med Res Methodol. 2018;18(1):143. doi:10.1186/s12874-018-0611-x 30453902 PMC6245623

[12] Peters MDJ , Marnie C , Colquhoun H , et al. Scoping reviews: reinforcing and advancing the methodology and application. Syst Rev. 2021;10(1):263. doi:10.1186/s13643-021-01821-3 34625095 PMC8499488

[13] Tricco AC , Lillie E , Zarin W , et al. A scoping review on the conduct and reporting of scoping reviews. BMC Med Res Methodol. 2016;16(1):15. doi:10.1186/s12874-016-0116-4 26857112 PMC4746911

[14] Church HR , Brown MEL , Govender L , Clark D . Beyond the bedside: protocol for a scoping review exploring the experiences of non‐practicing healthcare professionals within health professions education. Syst Rev. 2023;12(1):207. doi:10.1186/s13643-023-02364-5 37946279 PMC10633985

[15] Arksey H , O'Malley L . Scoping studies: towards a methodological framework. Int J Soc Res Methodol. 2005;8(1):19‐32. doi:10.1080/1364557032000119616

[16] Tricco AC , Lillie E , Zarin W , et al. PRISMA extension for scoping reviews (PRISMA‐ScR): checklist and explanation. Ann Intern Med. 2018;169(7):467‐473. doi:10.7326/M18-0850 30178033

[17] MacNeil M . From nurse to teacher: recognizing a status passage. J Adv Nurs. 1997;25(3):634‐642. doi:10.1046/j.1365-2648.1997.1997025634.x 9080292

[18] Seal J . Multiple professional identities: a personal exploration of the transition from nurse to lecturer. J Health Visit. 2017;5(2):94‐99. doi:10.12968/johv.2017.5.2.94

[19] McDermid F , Peters K , John Daly J , Jackson D . ‘I thought I was just going to teach’: stories of new nurse academics on transitioning from sessional teaching to continuing academic positions. Contemp Nurse. 2013;45(1):46‐55. doi:10.5172/conu.2013.45.1.46 24099225

[20] Neese R . A transformational journey from clinician to educator. J Contin Educ Nurs. 2003;34(6):258‐262. doi:10.3928/0022-0124-20031101-08 14650565

[21] Fox AS . The Experience of Nurse Faculty New to a Full Time Academic role and Intent to Stay in Academia. Widener University; 2017.

[22] Murray C , Stanley M , Wright S . The transition from clinician to academic in nursing and allied health: a qualitative meta‐synthesis. Nurse Educ Today. 2014;34(3):389‐395. doi:10.1016/j.nedt.2013.06.010 23827093

[23] Weidman NA . The lived experience of the transition of the clinical nurse expert to the novice nurse educator. Teach Learn Nurs. 2013;8(3):102‐109. doi:10.1016/j.teln.2013.04.006

[24] Grabanski JL . The Experiences of Occupational Therapy Clinicians Transitioning to the Role of Faculty Member: Implications for Faculty Development (Doctoral dissertation,. North Dakota State University; 2014.

[25] Lee SL , Rees CE , O'Brien BC , Palermo C . Identities and roles through clinician‐educator transitions: a systematic narrative review. Nurse Educ Today. 2022;118:105512. doi:10.1016/j.nedt.2022.105512 36054976

[26] Bustraan J , Dijkhuizen K , Velthuis S , et al. Why do trainees leave hospital‐based specialty training? A nationwide survey study investigating factors involved in attrition and subsequent career choices in the Netherlands. BMJ Open. 2019;9(6):e028631. doi:10.1136/bmjopen-2018-028631 PMC6589009 31175199

[27] Duffield C , Aitken L , O'Brien‐Pallas L , Wise WJ . Nursing: a stepping stone to future careers. JONA: J Nurs Adm. 2004;34(5):238‐245. doi:10.1097/00005110-200405000-00007 15167420

[28] Crist P . Career transition from clinician to academician: responsibilities and reflections. Am J Occup Ther. 1999;53(1):14‐19. doi:10.5014/ajot.53.1.14 9926213

[29] Govender L . Separation anxiety: walking away from clinical practice. Med Teach. 2023;45(2):229‐230. doi:10.1080/0142159X.2022.2130743 36214380

[30] Jamieson I , Taua C . Leaving from and returning to nursing practice: contributing factors. Nurs Prax NZ. 2009;25(2):15‐27. doi:10.36951/NgPxNZ.2009.003 19928648

[31] Schattner P , Klein B , Piterman L , Sturmberg J , McCall L . Impact of master of family medicine degree by distance learning on general practitioners' career options. Med Teach. 2007;29(4):e85‐e92. doi:10.1080/01421590701287905 17786737

[32] Siler BB , Kleiner C . Novice faculty: encountering expectations in academia. J Nurs Educ. 2001;40(9):397‐403. doi:10.3928/0148-4834-20011201-05 11769950

[33] Chargualaf KA , Elliott B , Patterson B . The transition from military nurse to nurse faculty: a descriptive study. Int J Nurs Educ Scholarsh. 2017;14(1). doi:10.1515/ijnes-2017-0027 29190213

[34] Axiak S , Axiak M . An exploration of the transition of clinical nurses to an academic nurse lecturer role. Nurs Educ Perspect. 2024;45(1):37‐39. doi:10.1097/01.NEP.0000000000001084 36584351

[35] Richards R , McLeod R , Latter D , et al. Toward late career transitioning: a proposal for academic surgeons. Can J Surg. 2017;60(5):355‐358. doi:10.1503/cjs.007617 28742011 PMC5608586

[36] Hunter J , Hayter M . A neglected transition in nursing: the need to support the move from clinician to academic properly. J Adv Nurs. 2019;75(9):1820‐1822. doi:10.1111/jan.14075 31115061

[37] Grassley JS , Strohfus PK , Lambe AC . No longer expert: a meta‐synthesis describing the transition from clinician to academic. J Nurs Educ. 2020;59(7):366‐374. doi:10.3928/01484834-20200617-03 32598005

[38] Wongpimoln B , Pholputta L , Ngernthaisong C , Sarnkhaowkhom C . Transitional experiences from clinical nurse experts to novice nurse lecturers in the university for local development in Thailand: a phenomenological study. Nurse Media J Nurs. 2021;11(2):197‐209. doi:10.14710/nmjn.v11i2.37366

[39] Beres J . Staff development to university faculty: reflections of a nurse educator. In: Nursing forum. Vol.41, No. 3. Blackwell Publishing Inc.; 2006:141‐145. doi:10.1111/j.1744-6198.2006.00050.x 16879149

[40] Ward M . Transition from practice to teaching: the advice I wish I received. J Nurs Educ. 2023;62(1):3‐4. doi:10.3928/01484834-20230118-01 36652584

[41] Goodrich RS . Transition to academic nurse educator: a survey exploring readiness, confidence, and locus of control. J Prof Nurs. 2014;30(3):203‐212. doi:10.1016/j.profnurs.2013.10.004 24939330

[42] Diekelmann N . New pedagogies for nursing: experienced practitioners as new faculty: new pedagogies and new possibilities. J Nurs Educ. 2004;43(3):101‐103. doi:10.3928/01484834-20040301-04 15072335

[43] Kreger MA . Down the Rabbit Hole: A Grounded Theory of Health Professionals' Transitioning from Practice to Academia (Doctoral dissertation). Allen College; 2019.

[44] Dempsey LM . The experiences of Irish nurse lecturers role transition from clinician to educator. Int J Nurs Educ Scholarsh. 2007;4(1):Article13. doi:10.2202/1548-923X.1381 17542779

[45] Duffy R . Nurse to educator? Academic roles and the formation of personal academic identities. Nurse Educ Today. 2013;33(6):620‐624. doi:10.1016/j.nedt.2012.07.020 22922027

[46] Aguayo‐González M , Weise C . Career transition and identity development in academic nurses: a qualitative study. J Constructivist Psychol. 2022;35(4):1371‐1389. doi:10.1080/10720537.2021.1936711

[47] Esper PS . Facing transition‐nurse clinician to nurse educator. J Nurs Educ. 1995;34(2):89‐91. doi:10.3928/0148-4834-19950201-12 7707145

[48] Hoffman DM . Transitional experiences: from clinical nurse to nurse faculty. J Nurs Educ. 2019;58(5):260‐265. doi:10.3928/01484834-20190422-03 31039259

[49] Anderson JK . The work‐role transition of expert clinician to novice academic educator. J Nurs Educ. 2009;48(4):203‐208. doi:10.3928/01484834-20090401-02 19441636

[50] Gould J . Metamorphosis: the journey from practice to education. Midirs Midwifery Digest. 2016;26(1):11‐14.

[51] Hurst KM . Experiences of new physiotherapy lecturers making the shift from clinical practice into academia. Physiotherapy. 2010;96(3):240‐247. doi:10.1016/j.physio.2009.11.009 20674657

[52] Culleiton AL , Shellenbarger T . Transition of a bedside clinician to a nurse educator. Medsurg Nurs. 2007;16(4):253‐257. 17907698

[53] Mirtz TA , Hebert JJ , Wyatt LH . Attitudes of non‐practicing chiropractors: a pilot survey concerning factors related to attrition. Chiropr Osteopat. 2010;18(1):29. doi:10.1186/1746-1340-18-29 21050461 PMC2992535

[54] Mukhalalati BA , Taylor A . Adult learning theories in context: a quick guide for healthcare professional educators. J Med Educ Curric Dev. 2019;6:2382120519840332. doi:10.1177/2382120519840332 PMID: 31008257; PMCID: PMC6458658 31008257 PMC6458658

[55] Morrissette PJ . Recruitment and retention of Canadian undergraduate psychiatric nursing faculty: challenges and recommendations. J Psychiatr Ment Health Nurs. 2011;18(7):595‐601. doi:10.1111/j.1365-2850.2011.01708.x 21848593

[56] Fain EA . Bridging the gap: helping more practitioners become academics. OT Practice. 2011;16(3):8‐12.

[57] Blackmer AB , Thompson AM , Jeffres MN , Glode AE , Mahyari N , Thompson M . Clinical pharmacy academic career transitions: viewpoints from the fieldPart 1: understanding feedback, evaluation, and advancement. Curr Pharm Teach Learn. 2018;10(2):123‐127. doi:10.1016/j.cptl.2017.10.017 29706264

[58] Cleary M , Horsfall J , Jackson D . Mental health nursing: transitions from practice roles to academia. Perspect Psychiatr Care. 2011;47(2):93‐97. doi:10.1111/j.1744-6163.2010.00280.x 21426354

[59] McDonald PJ . Transitioning from clinical practice to nursing faculty: lessons learned. J Nurs Educ. 2010;49(3):126‐131. doi:10.3928/01484834-20091022-02 19877571

[60] Danna D , Schaubhut RM , Jones JR . From practice to education: perspectives from three nurse leaders. J Contin Educ Nurs. 2010;41(2):83‐87. doi:10.3928/00220124-20100126-01 20166648

[61] Logan PA , Gallimore D , Jordan S . Transition from clinician to academic: an interview study of the experiences of UK and Australian registered nurses. J Adv Nurs. 2016;72(3):593‐604. doi:10.1111/jan.12848 26552602

[62] van Gennep, A . The Rites of Passage (Chicago, IL & London: The University of Chicago Press, 1960.

[63] Gordon L , Rees CE , Jindal‐Snape D. Doctors' identity transitions: Choosing to occupy a state of ‘betwixt and between’. Med Educ. 2020;54(11):1006‐1018. 32402133 10.1111/medu.14219

[64] Kerins J , Smith SE , Tallentire VR. ‘Just pretending’: Narratives of professional identity transitions in internal medicine. Med Educ. 2023;57(7), 627‐636. 36316289 10.1111/medu.14965

[65] Brown MEL, Proudfoot A, Mayat NY. A phenomenological study of new doctors’ transition to practice, utilising participant‐voiced poetry. Adv in Health Sci Educ. 2021;26:1229‐1253. 10.1007/s10459-021-10046-x PMC8452574 33847851

[66] Paulson N, Hernes T. Managing Boundaries in Organizations:Multiple Perspectives. London: Palgrave Macmillan, 2003.

[67] Ibarra H, Obodaru O. Betwixt and between identities: Liminal experience in contemporary careers. Research in Organizational Behavior, 2006;36:47‐64.

[68] Choudhry UK . Faculty practice competencies: nurse educators' perceptions. Can J Nurs Res Arch. 1992;5‐18. 1296870

[69] Aquino E , Lee YM , Spawn N , Bishop‐Royse J . The impact of burnout on doctorate nursing faculty's intent to leave their academic position: a descriptive survey research design. Nurse Educ Today. 2018;69:35‐40. doi:10.1016/j.nedt.2018.06.027 30007145

[70] Baker B , DiPiro JT . Evaluation of a structured onboarding process and tool for faculty members in a school of pharmacy. Am J Pharm Educ. 2019;83(6):7100. doi:10.5688/ajpe7100 31507295 PMC6718493

[71] Foley BJ , Redman RW , Horn EV , Davis GT , Neal EM , van Riper ML . Determining nursing faculty development needs. Nurs Outlook. 2003;51(5):227‐232. doi:10.1016/S0029-6554(03)00159-3 14569229

[72] Gazza EA , Shellenbarger T . Successful enculturation: strategies for retaining newly hired nursing faculty. Nurse Educ. 2005;30(6):251‐254. doi:10.1097/00006223-200511000-00009 16292147

[73] Mays KA , Burns LE , Branch‐Mays G , Quock R . Junior faculty perspectives on the academic environment: a call for development and onboarding. J Dent Educ. 2022;86(7):804‐813. doi:10.1002/jdd.12903 35181890

[74] NLN , 2018. https://www.nln.org/docs/default-source/uploadedfiles/professional-development-programs/healthful-work-environment-toolkit.pdf?sfvrsn=87d8da0d_0

[75] Daw P , Mills ME , Ibarra O . Investing in the future of nurse faculty: a state‐level program evaluation. Nurs Econ. 2018;36(2):59‐82.

[76] Dunham‐Taylor J , Lynn CW , Moore P , McDaniel S , Walker JK . What goes around comes around: improving faculty retention through more effective mentoring. J Prof Nurs. 2008;24(6):337‐346. doi:10.1016/j.profnurs.2007.10.013 19022206

[77] Schriner CL . The influence of culture on clinical nurses transitioning into the faculty role. Nurs Educ Perspect. 2007;28(3):145‐149. 17557636

[78] McMurtrie LJ , Cameron M , OLuanaigh P , Osborne YT . Keeping our nursing and midwifery workforce: factors that support non‐practising clinicians to return to practice. Nurse Educ Today. 2014;34(5):761‐765. doi:10.1016/j.nedt.2013.08.017 24080270

[79] Harms BA, Heise CP, Gould JC, Starling JR. A 25‐year single institution analysis of health, practice, and fate of general surgeons. Annals of surgery, 2005;242(4):520‐529. 10.1097/01.sla.0000184223.76854.29 PMC1402353 16192812

[80] Kenny G, Pontin D, Moore L. Negotiating socialisation: the journey of novice nurse academics into higher education. Nurse Education Today, 2004;24(8):629‐637. 10.1016/j.nedt.2004.08.002 15519446

